# Supplementary material for: Complexome profiling of the Chlamydomonas psb28 mutant reveals TEF5 as an early PSII assembly factor
Source: Plant Cell. 2025 Mar 17;37(6):koaf055. doi: 10.1093/plcell/koaf055 (PMC12164590; doi:10.1093/plcell/koaf055)
Supplement: koaf055_Supplementary_Data [file koaf055_supplementary_data.zip › psb28_interactive.zip!/plots/intensity/qualityPlots/peptides/PRPL7_Cre13.g581650.t1.2.html]

### Cre13.g581650.t1.2

Trivial name: PRPL7  
SeqCoverage [median]: 42.00  
Mass: 16.88 kDa  
  
 

| Sequence | MissedCleavage | Mass [Da] | wtIntensity | muIntensity | Score |
| --- | --- | --- | --- | --- | --- |
| AQLLEAGAK | 0 | 899.51 | 4335.84 | 14121.80 | 166.52 |
| ATPAVSEIVDKLK | 1 | 1369.78 | 0.00 | 8783.68 | 80.52 |
| TFGVDASAAAPVAMAAIAAPGAAAAPAVEEK | 0 | 2795.42 | 0.00 | 1556.37 | 45.27 |
| TTFDVVLEEIPADKK | 1 | 1703.90 | 1843.80 | 7698.62 | 82.55 |
|  |  |  | 6179.6 | 32160.5 |  |
